# Adverse Drug Reactions Associated With Drugs Prescribed in Psychiatry: A Retrospective Descriptive Analysis in a Tertiary Care Hospital

**DOI:** 10.7759/cureus.19493

**Published:** 2021-11-12

**Authors:** Sneha Ambwani, Siddhartha Dutta, Govind Mishra, Hina Lal, Surjit Singh, Jaykaran Charan

**Affiliations:** 1 Department of Pharmacology, All India Institute of Medical Sciences, Jodhpur, IND; 2 Department of Pharmacology, All India Institute of Medical Sciences, Bhubaneshwar, IND

**Keywords:** side effects, anti-depressants, anti-psychotics, psychiatry, pharmacovigilance, adverse drug reactions

## Abstract

Background

Psychiatric disorders are chronic in nature which often require long and continuous medications. These medications are known to cause adverse effects on their use. Their monitoring and prevention are crucial for the practicing family and community physicians.

Method

This is a cross-sectional retrospective study conducted to analyze all the spontaneous adverse drug reactions (ADRs) reported from the psychiatry department to the ADR Monitoring Center, Department of Pharmacology, AIIMS Jodhpur during the time period from 2014 to 2020.

Results

A total of 334 ADRs were reported. The majority of the ADRs were reported from antipsychotics (60.6%) followed by antidepressants (25.5%) and antiepileptic drugs (5.8%). On further subgroup analysis of the drug classes among antipsychotics, Clozapine (15.8%) was the leading offending agent. Similarly, among Antidepressants, Escitalopram (6.1%) was causing the most side effects. The most common ADR reported was sedation (7.26%) followed by salivary hypersecretion (6.7%), akathisia (5.52%), and weight gain (5.52%).

Conclusion

Knowledge of common ADRs help in better management of the diseases and psychotropics as a class has various frequents ADRs. Early detection and suitable intervention can help the community physicians in the proper care of the patients and rational use of drugs.

## Introduction

Drugs, which have an indispensable role in treating diseases or ailments, while treating the patients sometimes may have some side effects [1]. Adverse event and adverse effect/adverse drug reaction are the two terms that are frequently used in the world of pharmacovigilance and are misunderstood and confused with each other quite often. The World Health Organization (WHO) has defined adverse drug reaction (ADR) as ‘A response to a drug which is noxious and unintended, and which occurs at doses normally used in man for the prophylaxis, diagnosis, or therapy of disease, or for the modifications of physiological function’[2]. An adverse event has been defined as ‘Medical occurrence temporally associated with the use of a medicinal product, but not necessarily causally related’[2]. Monitoring of these occurrences is clubbed under pharmacovigilance and is defined as ‘The science and activities relating to the detection, assessment, understanding, and prevention of adverse effects or any other possible drug-related problems’ [3]. When the world was shaken by the thalidomide tragedy, the need for monitoring of the approved drugs came to light [4]. Continuous monitoring of the drugs even after approval has led to uncovering of serious side effects which has resulted in many black box warnings and even withdrawal of the implicated drugs [5-7]. COX-2 selective NSAIDs such as rofecoxib and valdecoxib have been withdrawn due to their cardiovascular complications [8]. Other examples of drugs withdrawn in recent years are rosiglitazone, rimonabant, cisapride, astemizole, nimesulide in children, etc [9]. Pharmacovigilance plays an important role in signal detection, generation, and confirmation of a new ADR [10]. In India, the PvPI (Pharmacovigilance Programme of India) under IPC (Indian Pharmacopoeia Commission) as the national coordination Centre collect, analyze, generate signals and distribute the information to all stakeholders such as the Central Drugs Standard Control Organisation (CDSCO), the Uppsala Monitoring Centre (WHO), pharmaceutical industries, health professionals, ADRs Monitoring Centre (AMC), National Immunization Programme, etc [11,12].

To date, 311 AMCs have been set up all over India to be the first contact destination for any type of ADR reporting [13]. In the analysis of serious adverse events from 2006 to 2014 reported to FDA, anti-neoplastic drugs were most commonly associated with adverse event-related death. Three anti-depressants (Paroxetine, Fluoxetine, Sertraline) and one anti-psychotic (Quetiapine) has been ranked among the top 10 drugs causing disability [14].

Psychotropic drugs have a direct effect on the delicate balance of neurotransmitters controlling the behavior and functioning of the brain. Any deviation in the balance can lead to unwanted and unprecedented behavioral changes although various other factors also contribute to the neurobehavioral changes [15]. A range of 0.69%- 10.2% has been reported as the incidence of ADRs with drugs used in psychiatry in previous studies [16-19]. Sengupta et al. in a study in 2011 found antipsychotics followed by SSRIs (selective serotonin reuptake inhibitors and lithium caused major adverse events in the patients [20]. Extrapyramidal side effects, weight gain, somnolence, akathisia, sialorrhea, constipation, dry mouth, sexual dysfunction are some of the vast side effects that are reported by patients on different psychotropic medication such as antipsychotics, antidepressants, antimanic drugs, etc [21-23] These ADRs can force a patient to be non-adherent or discontinue the therapy which further increases the burden of psychiatric illness [24]. There is a reluctance to seek out help for a mental health problem due to social stigma in the society. ADRs not only increase the duration of hospital stay but also increase the cost of treatment due to the extra care needed to negate the effect of adverse effects due to the therapy. This might further increase the hesitancy among the patients [25]. About 0.2%-6.0% of the healthcare budget is being utilized to tackle the problem of the adverse effect produced by different medications [26]. Since psychotropic medications are among the top rankers for causing side effects, their pattern would help in understanding and preventing adverse effects in the local environment. Since there is a deep impact of ADRs on public health both economically and socially, this study was planned to evaluate the pattern of adverse effects experienced by patients suffering from psychiatric ailments over a period of years (2014-2020) in a tertiary care hospital.

## Materials and methods

This is a cross-sectional retrospective study conducted to analyze all the ADRs reported from the psychiatry department to the ADR Monitoring Center, Department of Pharmacology, AIIMS Jodhpur. The ADRs were spontaneously reported to the AMC. The ADRs reported during the time period from 2014 to 2020 for the medications used for the psychiatric patients were taken into account for analysis. The data was segregated by different parameters such as age, offending agent, dose, type of reaction, indication, outcome, causality, etc. The causality assessment of the reported ADRs was done based on the WHO UMC scale.

The Medical Dictionary for Regulatory Activities (MedDRA) classification which was developed by the International Council for Harmonisation of Technical Requirements for Pharmaceuticals for Human Use (ICH) was used to assess the system-wise distribution of the ADRs based on System Organ Classes (SOCs) and Preferred Terms (PTs) [27,28].

The data were entered in Microsoft Excel 16 and analyzed using descriptive statistics. The data are presented as frequencies and percentages. The study was approved by the institutional ethics committee with certificate number AIIMS/IEC/2020/3248.

## Results

**Figure 1:**
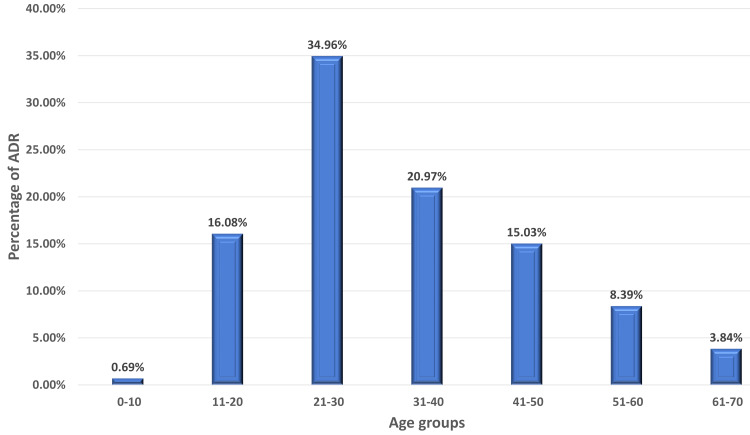
Age distribution of ADRs reported from psychiatric patients. ADRs: adverse drug reactions.

**Figure 2:**
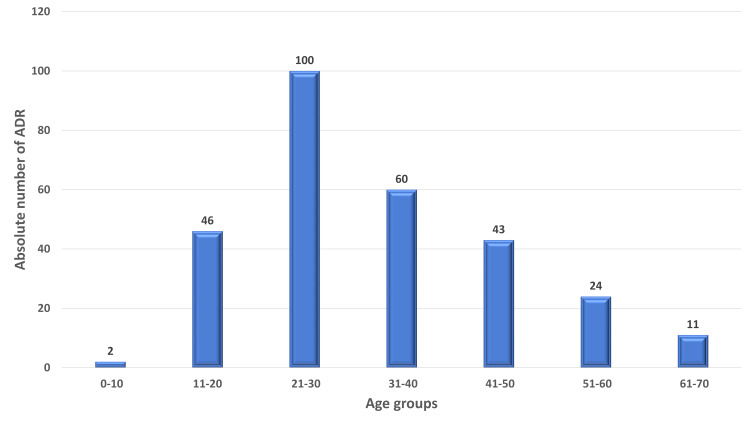
Numerical distribution of ADRs according to age group. ADRs: adverse drug reactions.

**Figure 3:**
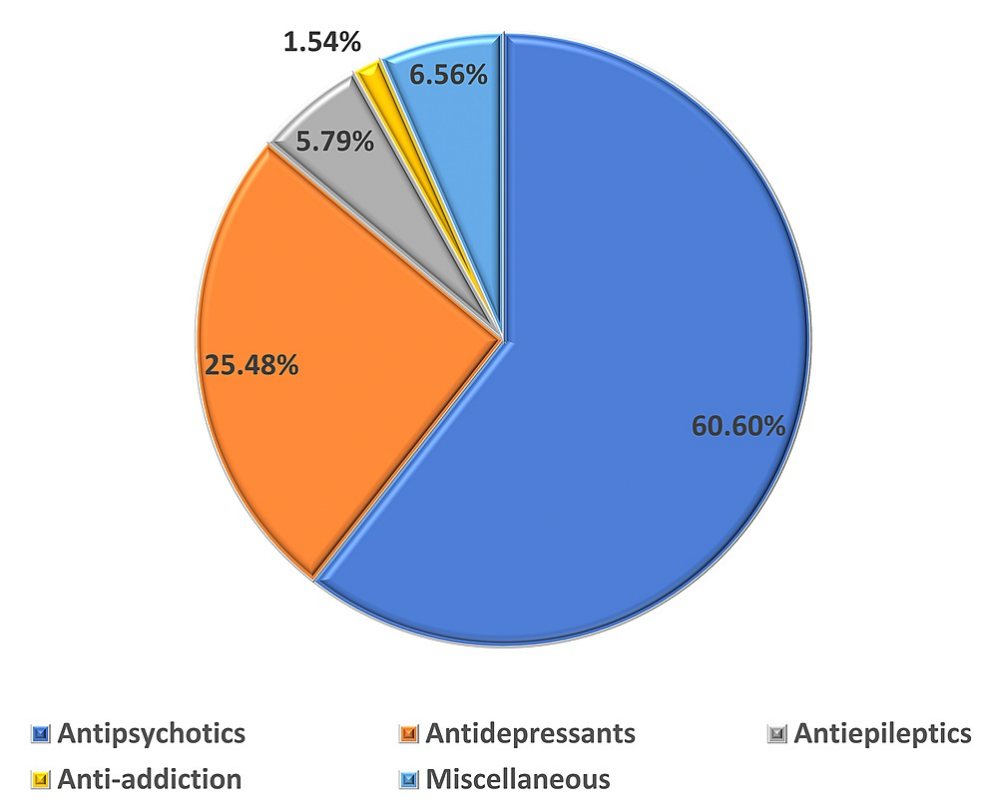
Distribution of ADRs caused by various classes of drugs. ADRs: adverse drug reactions.

A total of 334 Adverse events were reported from 286 patients from the psychiatry department. The mean age of the patients was 33.4 ± 13(SD) and had almost equal representation from either gender. About 73% of the ADRs were reported from the age group 19-45 years (Table 1) and the majority were reported in the age group of 21-30 years (35%) and 31-40 years (21%) (Figures 1, 2). Of 334 reported ADRs, 259 ADRs were reported from a single drug, and 27 were with a combination of drugs used in psychiatric and neurological disorders. The majority of the ADRs were reported from antipsychotics (60.6%) followed by antidepressants (25.5%) and antiepileptics (5.8%) (Figure 3). On further subgroup analysis of the drug classes, a bulk of ADRs among antipsychotics was reported with the use of Clozapine (15.8%), Olanzapine (12%), and Haloperidol (10.4%). Similarly, among Antidepressants, Escitalopram (6.1%), Mirtazapine (3.4%), Desvenlafaxine (3.4%), and in antiepileptics, Valproate (2.3%) and Carbamazepine (1.1%) were commonest (Table 2). Among the drugs used in combination(N=27), Quetiapine(N=8) and Valproate (N=7) were the most commonly used drugs along with various other drugs which led to the ADRs (Table 3).

**Table 1:** Basic demographic data.

Parameter		Frequency (n=286)
Gender	Males	139 (48.6%)
	Females	147 (51.4%)
Age	0-18	27 (9.44%)
	19-45	209 (73.07%)
	46-60	39 (13.63%)
	61 and above	11 (3.84%)

**Table 2:** Distribution of various classes of drugs causing ADRs in patients treated in the psychiatry department. ADR: adverse drug reaction.

Drug class	Individual drugs	No of cases (n=259)	Percentage
Anti-Psychotic	Clozapine	41	15.83
	Olanzapine	31	11.96
	Haloperidol	27	10.42
	Risperidone	14	5.40
	Aripiprazole	13	5.01
	Amisulpride	10	3.86
	Quetiapine	9	3.47
	Lurasidone	3	1.15
	Chlorpromazine	2	0.77
	Levosulpiride	2	0.77
	Ziprasidone	2	0.77
	Fluphenazine	2	0.77
Antidepressants	Escitalopram	16	6.17
	Mirtazapine	9	3.47
	Desvenlafaxine	9	3.47
	Clomipramine	8	3.08
	Amitriptyline	6	2.31
	Fluoxetine	6	2.31
	Sertraline	5	1.93
	Nortriptyline	2	0.77
	Fluvoxamine	1	0.38
	Duloxetine	1	0.38
	Dosulepin	1	0.38
	Paroxetine	1	0.38
	Duloxetine	1	0.38
	Trazodone	1	0.38
Anti-epileptics	Valproate	6	2.31
	Carbamazepine	3	1.15
	Levetiracetam	2	0.77
	Lamotrigine	1	0.38
	Divalproex	1	0.38
	Phenytoin	1	0.38
	Topiramate	1	0.38
Anti-addiction	Disulfiram	2	0.77
	Clonidine	1	0.38
	Naltrexone	1	0.38
Miscellaneous	Lithium	3	1.15
	Baclofen	3	1.15
	Flunarizine	3	1.15
	Lorazepam	1	0.38
	Zolpidem	1	0.38
	Naproxen	1	0.38
	Trihexyphenidyl	1	0.38
	Vitamin B12	1	0.38
	Propofol	1	0.38
	Atomoxetine	1	0.38
	Amantadine	1	0.38

**Table 3:** Distribution of various combinations of drugs causing ADRs in patients treated in the psychiatry department. ADR: adverse drug reaction.

Drug	Additional suspected drug used in combination	Frequency (N=27)
Quetiapine (N=8)	Fluoxetine	1
	Olanzapine	1
	Haloperidol	1
	Valproate	2
	Risperidone	1
	Amisulpride	1
	Aripiprazole	1
Valproate (N=7)	Olanzapine	2
	Sertraline	1
	Haloperidol	1
	Valproic acid	1
	Trihexyphenidyl	1
	Escitalopram+Resperidone	1
Dosulepin (N=3)	Venlafaxine	1
	Desvenlafaxine	1
	Methylcobalamin	1
Risperidone (N=3)	Haloperidol	1
	Loxapine	1
	Trineurosol inj.	1
Escitalopram (N=2)	Clonazepam	1
	Sertaline	1
Olanzapine (N=1)	Lorazepam	1
Gabapentin (N=1)	Amitriptyline	1
Lurasidone (N=1)	Chlorpromazine	1
Amisulpiride (N=1)	Aripiprazole	1

On analyzing the causality of the reported ADRs, majority were found to be under ‘possible’ (60.83%) followed by ‘probable’ (36%) category. The analysis of the outcomes of the reported ADRs showed that the majority of the patients were recovered (46.15%) or recovering (28.67%). By and large, the ADRs were not serious (98.25%) in nature, and hospitalization was required for only 1.74% of the patients (Table 4). The most common ADR reported was sedation (7.26%) followed by salivary hypersecretion (6.7%), akathisia (5.52%), and weight gain (5.52%) (Table 5).

**Table 4:** Assessment of various parameters associated with ADRs reported from psychiatric patients. ADR: adverse drug reaction.

Parameters		No of ADRs (n=286)	Percentage
Causality	Certain	9	3.14
	Probable	103	36.01
	Possible	174	60.83
Outcome	Recovered	132	46.15
	Recovering	82	28.67
	Not recovered	72	25.17
Seriousness	Not serious	281	98.25
	Hospitalization/prolonged	5	1.74

**Table 5:** Distribution of common ADRs in psychiatric patients. ADR: adverse drug reaction.

ADRs	No of events (%)
Sedation	25 (7.26)
Salivary hypersecretion	23 (6.68)
Akathisia	19 (5.52)
Weight increased	18 (5.23)
Hyperprolactinemia	16 (4.65)
Constipation	15 (4.36)
Tremor	12 (3.48)
Oculogyric crisis	10 (2.90)
Restlessness	10 (2.90)

The ADRs reported were further classified according to MedDRA classification based on System Organ Class and it was observed that to a large extent ADRs were associated with the nervous system disorder (32%), followed by gastrointestinal disorders (18.31%) and endocrine disorders (7.55%) (Table 6).

**Table 6:** System-wise MedDRA classification of reported ADRs in psychiatric patients. N = number of patients, n= number of adverse drug reactions (ADRs). MedDRA: Medical Dictionary for Regulatory Activities.

System Organ Class (SOC)	No of ADRs (N=286, n=334)	Percentage
Blood and lymphatic system	11	3.19
Cardiac disorders	5	1.45
Endocrine disorders	26	7.55
Eye disorders	10	2.90
Gastrointestinal disorders	63	18.31
General disorders	12	3.48
Infections and infestations	1	0.29
Investigations	20	5.81
Metabolism and nutrition disorders	5	1.45
Musculoskeletal and connective tissue disorder	9	2.61
Nervous system disorder	110	31.97
Psychiatric disorder	23	6.68
Renal and urinary disorder	6	1.74
Reproductive system and breast disorder	14	4.06
Respiratory, thoracic and mediastinal disorder	2	0.58
Skin and subcutaneous disorders	23	6.68
Vascular disorders	4	1.16

## Discussion

The current study was done to assess the ADRs reported by the psychiatry department. Majorly the ADRs were reported from the working-age group and almost equally from both genders. Antipsychotics, antidepressants, and antiepileptics were the leading drug classes causing the ADRs and among these classes Clozapine, Escitalopram and Valproate respectively were the commonest drugs found responsible. The majority of the ADRs were classified under possible category and had outcome as recovered. Maximally the ADRs were non-serious in nature and the most common ADRs reported were sedation, salivary hypersecretion, akathisia, and weight gain.

In this study, the gender distribution for the ADRs reported was almost similar with 48.6% males and 51.4% females. Previous studies conducted by Lucca et al. and Lakshmi Prasanna et al. reported similar results with females being 53.8% and 53.6% respectively [29,30]. On the contrary, a study by Barvaliya et al. showed the ADRs reported were more by males (57.3%) as compared to females [19].

The mean age observed in this study was 33.4 years which is comparable to Sridhar et al. (36.15) and Lucca et al. (35.6) [21,29]. The major age group with ADRs were the patients from the adult population with minor representation from the pediatric and elderly population. Age group 19-45 experienced the major share of the ADRs (73.07%) especially in the age group of 21-30 years claiming 34.96% of the share. Lucca et al. observed a similar observation in the age group 19-29 years (37.32%) [29]. Hotha et al. and Lakshmi Prasanna et al. in their study showed that the ADRs with psychiatric drugs are more common in the age group 37-54 years (48.61%) and 31-45 years (36%), respectively [30,31]. The probable reason for more reported ADRs from this group may be due to more prevalence of psychiatric illness in this particular age group as observed in the National Mental Health Survey of India 2015-2016 [19,32].

In the current analysis, antipsychotics were the major offending drug group with clozapine as the front-runner drug followed by olanzapine and haloperidol. The findings of the study were in line with the study conducted by Sridhar et al. and Sengupta et al. where the majority of ADRs were associated with atypical antipsychotic drugs [20,21] The findings were supported by Hotha et al., however, the primary offending drug was risperidone instead of clozapine [31]. Lucca et al. reported olanzapine to be the most common drug among antipsychotics causing ADRs [29]. Among antidepressants, which was the second commonest category in this study, escitalopram followed by mirtazapine and desvenlafaxine were common drugs causing. In support of our findings, escitalopram was reported as the primary culprit for ADRs by Sridhar et al. and Patel et al. [21,33]. Fluoxetine was reported by Barvaliya et al. as the most common drug causing ADRs [19].

The most common ADR reported among drugs used in psychiatric disorders was sedation followed by salivary hypersecretion, akathisia, and weight gain. Frequency of common ADRs was reported differently in various studies such as extrapyramidal side effects by Sharma et al., tremors by Hotha et al., weight gain by Sridhar et al. and Lucca et al. as the most common ADRs reported whereas dyskinesia, constipation, rigidity, slurring of speech, etc were other concomitant ADRs reported [21,22,29,31].

Causality assessment is an essential step in determining the relationship between ADR and suspected drugs, taking into consideration other factors for causing the ADR. Recovery of a patient can help establish a temporal relationship between the suspected drug and adverse events. In this study number of fully recovered patients was higher than those for recovering and not recovered. When causality assessment of these ADRs was done as per the WHO UMC causality assessment scale, 60.83% of ADRs fell into the possible category followed by 36.01% in probable and 3.14% in a certain category. This observation is supported by several previous studies conducted on psychiatric medications [19,31,34].

MedDRA is dedicated and standardized terminology adopted to overcome obstacles faced in converting medical events explained in different local terminology used in various parts of the world, making the data easily interpretable all over the world [35]. The study data indicated adverse events that were commonly associated with psychiatric drugs involved major organ systems like the nervous system (31.97%), followed by the gastrointestinal system (18.31%) according to MedDRA classification. A similar ADR profile was reported in other studies with the nervous system being the most commonly affected system which supported our observation [19,31].

Limitations

One major limitation of our study is that we were not able to check the frequency of administration of different antipsychotics to patients. As it was a retrospective analysis of data of ADRs reported to our center by the physicians, we were able to perform a descriptive analysis of ADR reported by medications prescribed in recommended doses for treatment of psychiatric illnesses.

## Conclusions

The pharmacovigilance programme tends to generate the signals for a rare and unknown adverse event but knowing the commonly faced scenarios in the case of ADRs is equally necessary to help the physicians in rational prescribing of the drug while keeping in mind the adverse event it can cause. The knowledge of ADRs produced by psychoactive drugs can help in better and early recognition of the adverse event. This can help the treating physicians to look for the warning signs and practice caution while prescribing the implicated drugs to reduce the overall burden of ADRs.

Recommendation and key messages:

The adverse drug events (ADEs) are any untoward medical occurrence that may show up during treatment with a drug but does not necessarily always have a causal relationship with the treatment.

The complete spectrum ADEs associated with the drugs are not completely known when a new drug is approved by the regulatory authorities and hence post marketing monitoring and reporting of these adverse events is crucial.

 The rare, unexpected and unknown adverse reactions are often encountered when the ADE monitoring is effectively done during the widespread use of the drug post approval

These collective aggregation of ADEs can lead to the generation of new signals for a drug which is highly essential to assess the safety of the drug for its use in the population in the long term

This study assessed the Adverse Drug Reactions (ADRs) associated with various drugs used in the psychiatry department.

There was an equal frequency of ADRs among males and females with the highest frequency among adults.

About 61% of drugs have a possible association with the ADRs reported. This is due to the fact that the majority of the drugs were prescribed as combination therapy.

Ninety-eight percent of ADRs were not serious in nature.

As per MedDRA classification, gastrointestinal and endocrine were the most common systems involved for the ADRs that were reported.

Physicians, nursing officers, other healthcare workers and patients should be vigilant with regard to any ADRs observed and report it to the earliest so that appropriate steps can be taken to mitigate the harm caused to the patient and prevent further damage which would help in the rational and safe use of drugs in therapeutics.
